# Tunnelized Facial Artery Myomucosal Island Flap: A Modification of the FAMM Flap that Enhance its Reconstructive Versatility

**DOI:** 10.1007/s12070-023-04169-3

**Published:** 2023-08-29

**Authors:** Luigi Angelo Vaira, Olindo Massarelli, Jerome R. Lechien, Carlos M. Chiesa-Estomba, Tareck Ayad, Giacomo De Riu

**Affiliations:** 1 Maxillofacial Surgery Unit, Department of Medicine, Surgery and Pharmacy, University of Sassari, Viale San Pietro 10, 07100 Sassari, Italy; 2 Biomedical Science Department, PhD School of Biomedical Science, University of Sassari, Sassari, Italy; 3 Maxillofacial Surgery Operative Unit, Department of Mental Health and Sense Organs, University Hospital of Siena, Santa Maria Le Scotte, Siena, Italy; 4 Department of Laryngology and Bronchoesophagology, Mons School of Medicine, UMONS. Research Institute for Health Sciences and Technology, EpiCURA Hospital, University of Mons (UMons), Mons, Belgium; 5 Department of Otolaryngology-Head Neck Surgery, Elsan Polyclinic of Poitiers, Poitiers, France; 6 Department of Otorhinolaryngology-Head & Neck Surgery, Hospital Universitario Donostia, San Sebastian, Spain; 7 Division of Otolaryngology-Head and Neck Surgery, Centre Hospitalier de l’Université de Montréal (CHUM), Université de Montéal, Montreal, Quebec Canada

**Keywords:** Tongue reconstruction, Buccinator myomucosal flap, Flap, Cheek myomucosal flap, FAMM, t-FAMMIF, Facial artery myomucosal island flap, Maxillofacial surgery, Otorhinolaryngology

Dear Editor,

We read the article by Lakhera et al. [1], reporting the use of buccinator myomucosal flaps for reconstructing small and medium-sized oral defects. These flaps represent a reliable and safe reconstructive technique that allows, unlike any other, a like-with-like reconstruction of the defect. This is of great importance considering that the mucosa of the oral cavity has unique characteristics, essential for the proper manifestation of a large number of vital functions that significantly influence an individual’s quality of life. In this regard, buccinator myomucosal flaps have previously demonstrated adequate functional reconstruction capabilities including an excellent sensory recovery and a low tendency for scar retraction [2].

We would like to make a few observations based on our own experience. First, it should be specified that the flap used by the authors is not the FAMM flap proposed by Pribaz et al. in 1992. The FAMM is an axial flap based on the facial artery During its harvesting, the facial vessels are not dissected to their origin in the neck, but a mucosal pedicle is maintained to ensure venous drainage. This reduces the versatility and rotation arc of the flap, which cannot be tunneled in the neck, limiting its use in dentate individuals, and requiring secondary pedicle sectioning. The flap used by the authors is an island flap, strictly pedicled on the facial vessels as described by Zaho et al. in 2003. This type of flap allows virtually the entire cheek to be harvested with vascularization guaranteed by the dense vascular network stretched between the facial and the buccal arteries systems. The tunnelized Facial Artery Myomucosal Island Flap (t-FAMMIF) was introduced by Massarelli et al. in 2008 and increases the flap’s rotation arc to reach contralateral sites, allows its use in dentate subjects, and avoids the second stage of pedicle sectioning [3].

Three cases of venous congestion are reported by the authors, leading to one case of total flap necrosis, all occurring when the facial vein was accidentally sectioned. The possibility of harvesting island myomucosal flaps pedicled solely on the facial artery, where venous drainage is ensured by venae comitantens, has already been described but is fraught with a very high rate of flap loss if the artery is dissected to its origin. In these cases, the artery dissection should stop at the mandibular body level without proceeding to the neck. Excessive dissection significantly increases the rate of venous return insufficiency and flap loss [4].

Finally, the authors included patients who underwent therapeutic neck dissection for the presence of lymph node metastases. Preserving the facial vessels in the neck is oncologically safe in patients with cN0 neck [5]. However, the presence of clinically evident lymph node metastases is a relative contraindication to preserving the facial vessels, and therefore to the harvesting of myomucosal flaps based on this vascular system, especially for level I positive nodes. In these cases, it is still possible to use myomucosal flaps pedicled on the buccal vessels, albeit sacrificing some versatility and rotation arc.
